# Development of ^177^Lu-phytate Complex for Radiosynovectomy

**Published:** 2013-05

**Authors:** Hassan Yousefnia, Amir Reza Jalilian, Ali Bahrami-Samani, Mohammad Mazidi, Mohammad Ghannadi Maragheh, Fereydoun Abbasi-Davani

**Affiliations:** 1 Radiopharmaceutical Research and Development Lab, Nuclear Science and Technology Research Institute (NSTRI), Tehran, Iran, P.O.Box:14395-836; 2 Faculty of Energy Engineering and Physics, Amirkabir University of Technology. Tehran, Iran; 3 Radiation Application Group, Faculty of Nuclear Engineering, Shahid Beheshti University, Tehran, Iran

**Keywords:** Biodistribution, Lutetium-177, Phytate Radiosynovectomy

## Abstract

***Objective(s):*** In this work a new possible agent for radiosynovectomy has been targeted for articular pain palliation.

***Materials and Methods:*** Lu-177 of 2.6-3 GBq/mg specific activity was obtained by irradiation of natural Lu_2_O_3 _sample with thermal neutron flux of 4 × 10^13^ n.cm^-2^.s^-1^. The product was converted into chloride form which was further used for labeling of ^177^Lu-phytate complex and checked using ITLC (MeOH: H_2_O: acetic acid, 4: 4: 2, as mobile phase). The complex stability and viscosity were checked in the final solution up to seven days. The prepared complex solution (100 µCi/100 µl) was injected intra-articularly to male rat knee joint. Leakage of radioactivity from injection site and its distribution in organs were investigated up to seven days.

***Results:*** The complex was successfully prepared with high radiochemical purity (>99.9 %). Approximately, the whole injected dose has remained in injection site seven days after injection.

***Conclusion:*** The complex was proved to be a feasible agent for cavital radiotherapy in oncology and rheumatology.

## Introduction

With the aging of the human population around the world, the need for the management of elderly-diseases such as rheumatoid arthritis and other joint problems has emerged. Also a majority of diseases can cause arthropathy leading to the pain, inflammation and also immobility of the patients such as spondyloarthropathy, Lyme disease, Behcet´s disease, persistent synovial effusion, haemophilic arthritis, calcium pyrophosphate dihydrate (CPPD) arthritis, pigmented villonodular synovitis (PVNS), persistent effusion after joint prosthesis, undifferentiated arthritis, etc (1). 

Radiosynovectomy (RSV) has been proposed as a potent palliative therapy around the world in the last two decades (1) and several radiopharmaceuticals have been developed for RSV including ^177^Lu-macroaggregates (2) and Ho-166 phytate complex (3).

Many beta-emitters such as ^153^Sm, ^177^Lu and ^166^Ho can be produced in reasonable amounts using (n, gamma) reactions. Owing to lutetium-177 suitable decay characteristics [T_1/2_ = 6.73 d, E_max_ = 497 keV, E = 112 keV (6.4%), 208 keV (11%)] as well as the feasibility of large-scale production in adequate specific activity and radionuclidic purity using a moderate flux reactor, ^177^Lu has been considered as a promising radionuclide for developing therapeutic radiopharmaceuticals.

Thus, various agents have been developed and used in therapy including ^177^Lu-labeled compounds, such as somatostatin receptor ligands (4), monoclonal antibodies (5), pain palliation compounds (6) and radiosynovectomy agents (7, 8).

**Figure 1:**
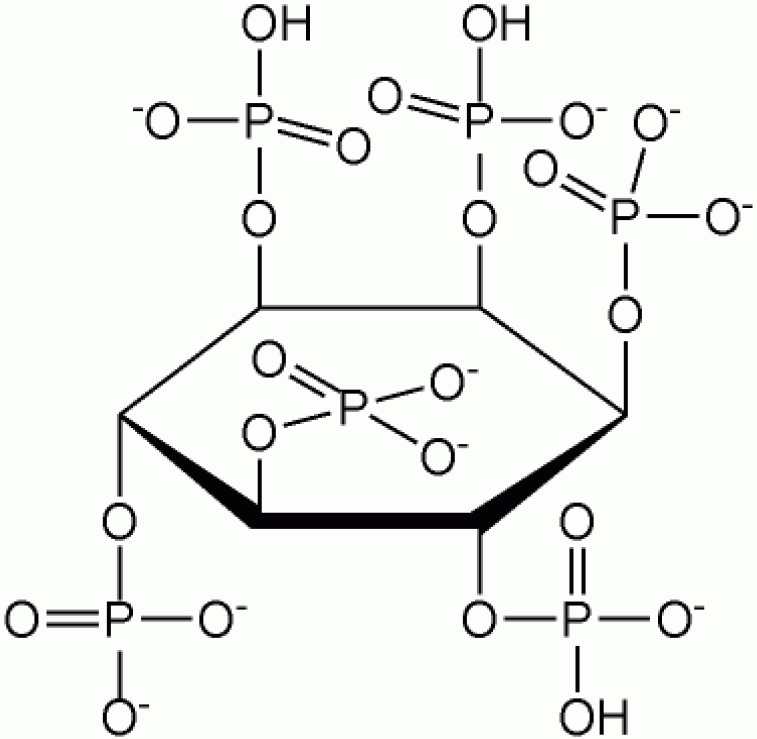
Chemical formula for phytate

Phytate, a salt form of inositol hexaphosphate (Figure 1), is the principal storage form of phosphorus in many plant tissues that chelates to many bi/tri-valent metals forming insoluble compounds. This compound has been widely used in nuclear medicine in complex form for diagnostic and therapeutic applications. 

In this research, ^177^Lu-Phytate complex production is described in details, followed by determination of complex radiochemical purity, stability, bio-distribution and imaging studies (after intra- articular and intravenous injection) in wild-type male rats. 

## Materials and Methods

^177^Lu was produced with a specific activity of approximately 70-80 mCi/mg and radionuclidic purity of 99.98% by irradiation of natural Lu_2_O_3_, targeted at a thermal neutron flux of approximately 4 10^13^ n/cm^2^.s for five days at Tehran Research Reactor (TRR). Phytate complex was prepared using a commercial phytate kit (Kavoshyar Co., Tehran, Iran, stannous chloride free). Chromatography paper, Whatman No. 1 was obtained from Whatman (Maidstone, UK). Radio-chromatography was performed by using a bioscan AR-2000 radio TLC scanner instrument (Bioscan, Washington, DC, USA). A high purity germanium (HPGe) detector coupled with a Canberra™ (model GC1020-7500SL) multichannel analyzer and a dose calibrator ISOMED 1010 (Dresden, Germany) were used for counting distributed activity in rat organs. All other chemical reagents were purchased from Merck (Darmstadt, Germany). Calculations were based on the 112 keV peak for ^177^Lu. All values were expressed as mean standard deviation (Mean SD) and the data were compared using student’s t-test. Statistical significance was defined as *P*<0.05. Animal studies were performed in accordance with the United Kingdom Biological Council's Guidelines on the Use of Living Animals in Scientific Investigations, 2nd edition. All of rats were purchased from Pasteur Institute of Iran, weighing 180-220 g (n=5) and were kept at routine day/night light program and were kept under common rodent diet pellets.

***Production and quality control of*** ^177^ ***Lu*** ***Cl***_3_ ***solution***

^177^Lu was produced by irradiation of natural Lu_2_O_3_ target (1 mg) at a thermal neutron flux of approximately 4 10^13^ n/cm^2^.s forfive days at the Tehran Research Reactor (TRR) according to reported procedures (9) in the Tehran Research Reactor. The irradiated target was dissolved in 200 µl of 1.0 M HCl, to prepare ^177^LuCl_3_ and diluted to the appropriate volume with ultra pure water, to produce a stock solution of final volume of 5 ml. The mixture was filtered through a 0.22 µm biological filter and sent for use in the radiolabeling step. For radionuclidic purity determination, the samples were checked by gamma-ray spectroscopy on an HPGe detector for 5 h basing on two major photons of ^177^Lu (6.4% of 0.112 MeV and 11% of 0.208 MeV). The radiochemical purity of the ^177^LuCl_3_ was checked using two solvent systems for ITLC (A: 10mM DTPA pH.4 and B: ammonium acetate 10%:methanol (1:1)).

*Synthesis of* ^177^ *Lu-phytate complex*

Briefly, 5 mCi (60 µg, 0.50 µl) of [^177^Lu]lutetium chloride acidic solution prepared above was transferred to a sterile borosilicate vial, and the mixture was evaporated using a flow of N_2_ gas and slight warming (50°C) for five min. Sterile normal saline solution (1 ml) was added to a commercial phytate kit (containing 10 mg phytic acid, no SnCl_2_) was added followed by vigorous shaking for 30 sec. The phytate mixture was then added in one portion to the activity-containing vial followed by stirring. The radiolabeling of the kit was checked by ITLC every ten min. After completion of the labeling, the mixture was filter-sterilized using 0.22 micron membrane.

*Quality control*

For measuring radiochemical purity and radiolabeling yield, a 1 μl sample of the [^177^Lu]lutetium phytate complex was spotted on a chromatography paper (Whatman No. 1), and developed in a mixture of methanol/water/acetic acid (4:4:2) as the mobile phase. 

*Stability testing of the radiolabeled compound in final formulation*

 Stability of ^177^Lu-phytate in final preparation was determined by storing the final solution at 4, 25 and 37°C for 7 days and performing frequent ITLC analysis to determine radiochemical purity. Also after subsequent ^177^Lu-labeling of the two month-stored kit, both labeling efficiency and radiochemical purity were determined.

*Biodistribution of* ^177^ *LuCl*_3_ *and [* ^177^ *Lu] lutetium phytate in male wild-type rats after intravenous injection*

To determine the biodistribution of free ^177^LuCl_3_ and [^177^Lu] lutetium phytate in case of any radioisotope/radiopharmaceutical leak from the injection site, the species dissolved in normal saline, were administered to wild-type rats. The animals were sacrificed by CO_2_ asphyxiation at selected times after injection (2-48 hr for free Lu^3+^). Dissection began by drawing blood from the aorta followed by removing heart, spleen, muscle, brain, bone, kidneys, liver, intestine, stomach, lungs and skin samples. For each animal, appropriate amount of ^177^LuCl_3_ or [^177^Lu] lutetium phytate activity (100-120 ±10 Ci, in 100 l,) was injected intravenously to rats through their tail vein. The animals were sacrificed at the exact time intervals and the specific activity of different organs was calculated as percentage of injected dose per gram using an HPGe detector.

*Biodistribution of [* ^177^ *Lu] lutetium phytate complex* *in* *wild-type rats after intra-articular administration*

To determine the accumulation of [^177^Lu] lutetium phytate in the intra-articular cavity their isotonic solutions were carefully administered to wild-type rats. A volume (100 l) of final radiolabeled solution containing 100-120 Ci radioactivity was injected intra-articular to rats. The animals were sacrificed at exact time intervals (2, 24, 120 and 168 hr). The specific activity of different organs was calculated as percentage of area under the curve of 112 keV peak per gram using an HPGe detector.

***Scintigraphic imaging of*** ^177^ ***Lu-phytate in wild-type rats***

For imaging studies, ^177^Lu-phytate solution (7.4 MBq, 200 l) was injected intravenously (through tail veins) and intra-articularly (through knee joint) to rats followed by propofol-xylazine mixture injection for anaesthetization. The images were acquired after administration of the radiopharmaceutical by a single-head SPECT system (Siemens, Germany) based on 112 keV peak (15% energy window). The rat-to-septa distance was 12 cm. 

## Results and Discussion

*Production and quality control of* ^177^ *Lu*

**Figure 2:**
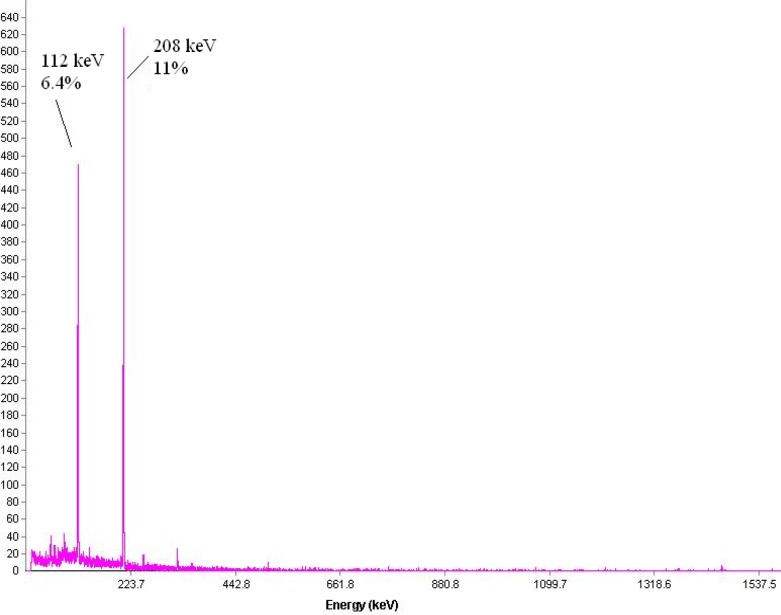
Gamma-ray spectrum for Lu-177 chloride solution used in this study

The radionuclide was prepared in a research reactor according to regular methods with a range of specific activity 2.6-3 GBq/mg for radiolabeling use. The obtained radionuclidic purity was 99.98% (Figure 2).

The radioisotope was dissolved in acidic media as a starting sample and was further diluted and evaporated for obtaining the desired pH and volume followed by sterile filtering. 

**Figure 3:**
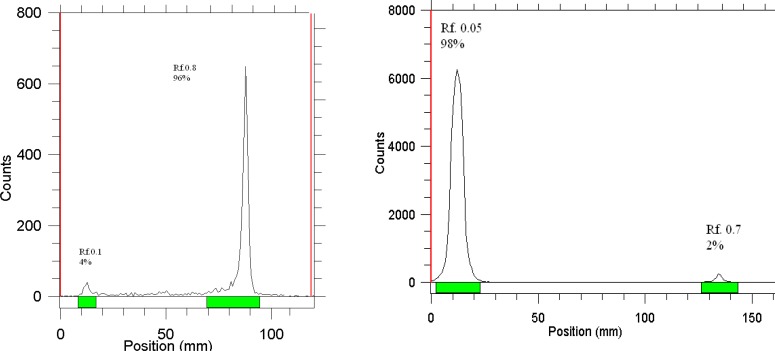
ITLC chromatograms of ^177^LuCl_3_ solution in DTPA solution (pH. 5) (left) and 10% ammonium acetate-methanol (1:1) solution (right) using Whatman no. 2

The radiochemical purity of the ^177^Lu solution was checked in two solvent systems, in 10mM DTPA, free Lu^3+^ cation is complexed to more lipophilic LuDTPA form and migrates to higher R_f_, while small radioactive fraction remains at the origin which could be related to other Lu ionic species, not forming LuDTPA complex, such as LuCl_4_^-^, etc. and/or colloids (Figure 3).

*Preparation of* *[* ^177^ *Lu* *]lutetium phytate* *complex*

The effects of various factors on the labeling yield of [^177^Lu]lutetium phytate were studied. In higher concentration, no significant difference exists on labeling yield for the added [^177^Lu]lutetium chloride activity (30 mCi). The phytate which had a molecular weight of 400 kDa was used to investigate the effect of phytate concentration on labeling yield at pH.3.5. 

Labeling yield increased with increasing phytate concentration and reached above 98% when the concentration reached 35 mg/3 ml. The highest labeling yield was achieved at pH=2.8-3.2 while decreased beyond this range. The labeling yield of 99% was achieved after 30 minutes. The effects of absence and presence of ascorbic acid (at various concentrations) as a complex stabilizer were also studied.

**Figure 4:**
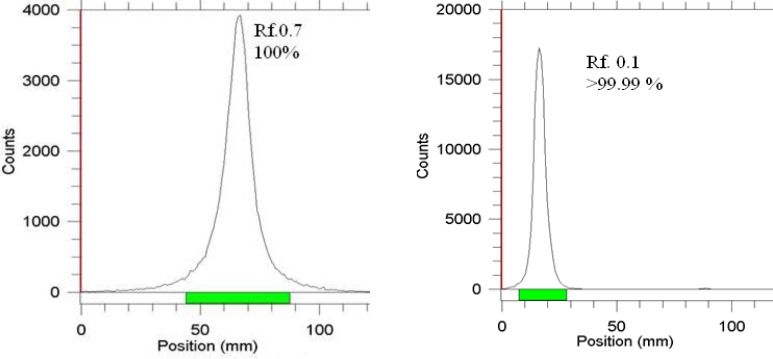
ITLC chromatograms of ^177^Lu-LuCl_3_ (left) and ^177^Lu-phytate solution (right) on Whatman no. 1 paper using methanol: water: acetic acid (4:4:2) mixture

ITLC using a mixture of methanol, water and acetic acid showed that the complex is majorly prepared in 30 min with 99% radiochemical purity; the remaining 1% is possibly attributed to other Lu ionic species which cannot react with phytate (Figure 4).

Based on the obtained results, the optimal procedure for the preparation of [^177^Lu]lutetium phytate complex with a high labeling yield is as follows. 35 mg of phytate (MW=400 kDa) was dissolved in 3.5 ml of 1% acetic acid aqueous solution. The acidity of obtained solution was adjusted to pH.3 by addition of 0.5 M NaOH solution and followed by the addition of [^177^Lu]lutetium chloride solution. Finally the total volume was adjusted to 4 ml by the addition of deionized water.

Stability studies of [^177^Lu]lutetium phytate complex

The stability of prepared [^177^Lu]lutetium phytate complex was checked up to 7 d after preparation. The complex was stable in acidic media (pH=3.5) and its radiochemical purity was above 99% even seven days after preparation. Also the stability of the complex was determined at 4°, 25 and 37°C for seven days and the data were almost consistent with the final solution stability.

***Biodistribution studies for free*** ^177^ ***Lu*** ***cation in rats***

**Figure. 5:**
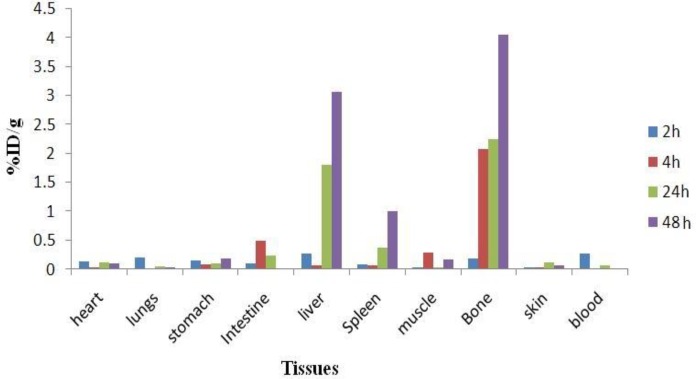
Percentage of injected dose per gram (ID/g %) of ^177^LuCl_3_ in wild-type rat tissues at 2, 4, 24 and 48 hr post injection

The animals were sacrificed by CO_2_ asphyxiation at selected times after injection (2, 4, 24 and 48 hr). Dissection began by drawing blood from the aorta followed by removing heart, spleen, muscle, bone, kidneys, liver, intestine, stomach, lungs and skin samples. The tissue uptakes were calculated as the percent of area under the curve of the related photo peak per gram of tissue (% ID/g) (Figure 5). The liver uptake of the cation is comparable to many other radio-metals mimicking ferric cation accumulation, about %3 of the activity accumulates in the liver after 48 hr. The transferrin-metal complex uptake and final liver delivery looks the possible route of accumulation. 

**Figure 6:**
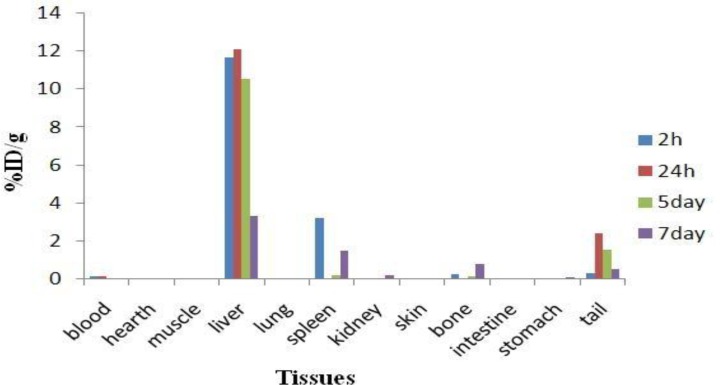
Percentage of injected dose per gram (ID/g %) of ^177^Lu-phytate in wild-type rat tissues at 2 hr, 24 hr, 5d and 7d post i.v. injection

**Figure 7:**
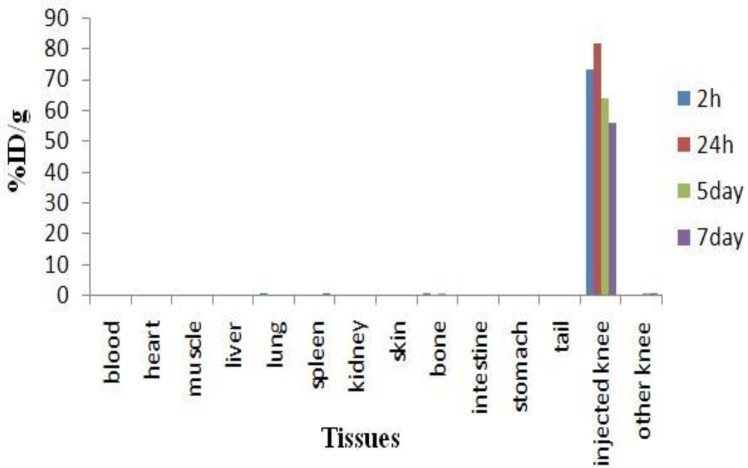
Distribution of [^177^Lu]-phytate in wild-type male rats, 4, 24, 120 hr and 168 h after intra-articular injection of 100 µCi of compound. % ID-percentage of injected dose. Each bar presents mean± SD (n=3)

The blood content is low at all time intervals and this shows the rapid removal of activity in the circulation. Lung, muscle and also skin do not demonstrate significant uptake while it is in accordance with other cations accumulation. A %4 bone uptake is observed for the cation which remains almost constant after 48 hr (data not shown). Spleen also has significant uptake possibly related to reticuloendothelial uptake. Kidney plays an important role in ^177^Lu cation excretion especially after 24 hr.

*Biodistribution studies after intravenous administration of* ^177^ *Lu-phytate in rats*

The distribution of injected dose in rat organs up to 144 h after intravenous injection of ^177^Lu-phytate chloride (60 µCi/100 µl) solution was determined for control studies. Based on these results, it was concluded that the most portion of injected activity of ^177^Lu-phytate was extracted to blood circulation and distributed in rat organs which was consistent with free Lu^3+^ distribution while administered intravenously (Figure 6).

**Figure 8:**
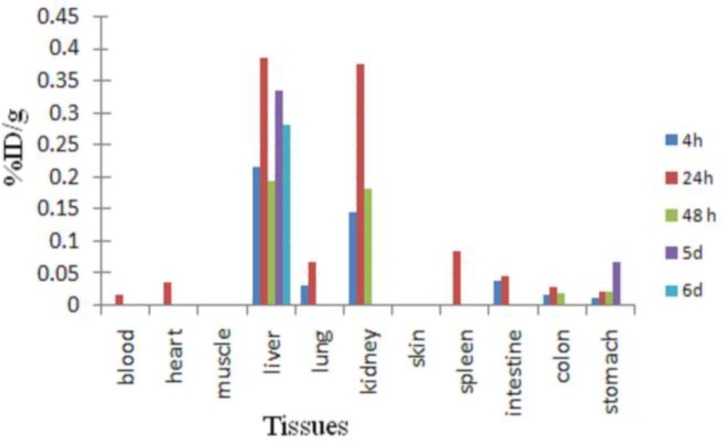
Distribution of [^177^Lu]-phytate in wild-type male rats excluding injected knee data at 4, 24, 48, 120 hr and 144 hr after intra-articular injection of 10 µCi of compound. % ID-percentage of injected dose. Each bar presents mean± SD (n=3)

*Biodistribution studies after intra-articular administration of* ^177^ *Lu-phytate cation in rats*

Figure 7 presents the distribution of injected dose in the rat organs at various time intervals after intra-articular injection of 100 µCi/100 µl of [^177^Lu]lutetium phytate complex as percentage of injected dose. In case of any leak from the joint, the complex would accumulate in reticuloendothelial (RE) system due to high molecular weight of the complex, unless the complex would dissociate at serum pH and Lu^3+^ cation would be formed.

Almost no detectable amounts of activity was observed in spleen and lung, which are two important RE organs, showing that no complex leak has occurred. Very negligible liver and kidney uptakes are observed which is possibly caused by ^177^Lu cation release from the injected joint and not the radiolabeled complex uptake. 

Figure 8 demonstrates the biodistribution of the compound among the tissues excluding the injected knee data in order to better understand the biodistribution of the leaks from the knee.

**Figure. 9:**
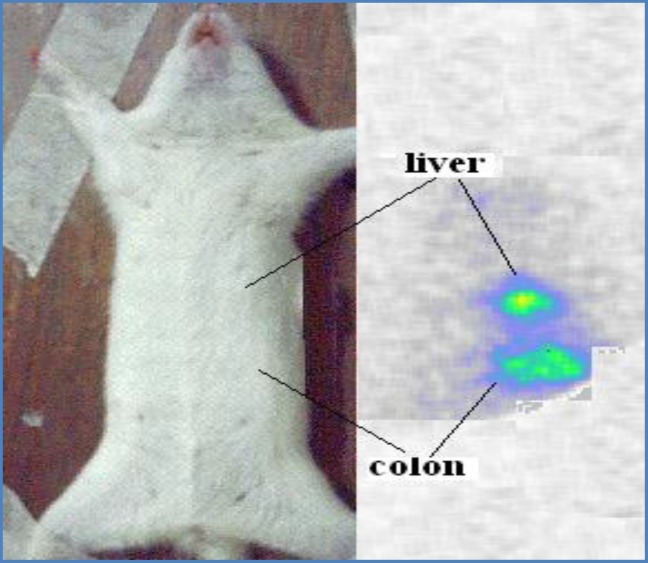
Scintigraphic images of ^177^Lu-phytate in wild-type rat tissues one week post i.v. injection

The distribution of the radioactivity among tissues after removing knee joint accumulation data demonstrated a typical Lu^3+ ^cation biodistribution among the tissues. It is believed that free Lu cation is the only radiochemical species escaping from the knee joint and no ^177^Lu-phytate complex was found in circulation.

For better visualization of the radiopharmaceutical sample, the compound was administered intravenously in to rat tail vein, and as expected the major radioactive content was found in the liver even after seven days, another major part of the activity was found in the colon due to the excretion of the compound and or possible metabolites (Figure 9).

The high liver accumulation of the compound suggests a possible route of administration of this radiopharmaceutical for hepatic malignancies specially hepatocellular carcinomas. Due to the accumulation and rather-long half-life beta emitter used, another preclinical study can be conducted on a suitable hepatic cancer animal model.

**Figure.10:**
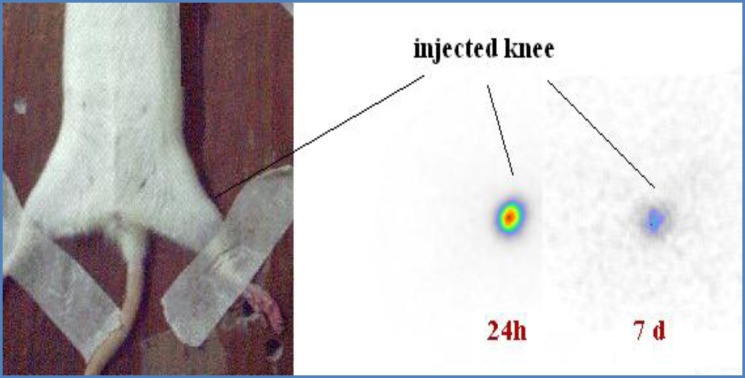
Scintigraphic images of ^177^Lu-phytate in wild-type rat knee 1 and 7 days post intra-articular injection

In order to observe the accumulation of the radioactivity in the injected knee joints, scintigraphic study was performed one and seven days post intra-articular injection of the radiopharmaceutical. As shown in the Figure 10, no detectable leak from the knee joint through surrounding tissues is observed.

## Conclusion

The [^177^Lu]Lutetium phytate complex was prepared with high radiochemical yield (>99 %) in the optimized condition. The prepared complex was stable in the final solution at room temperature, 37°C and presence of human serum, and can be used even seven days after preparation. Intra-articular injection of [^177^Lu]lutetium phytate complex to male wild-type rats and investigation of leakage of activity in the body showed that most of injected dose has remained in injection site 168 hr after injection by imaging and animal dissection studies. 

[^177^Lu]lutetium phytate is not only a possible radiosynovectomy agent for use in the clinics, but also can be a possible candidate for hepatic malignancy therapy when administered systemically.
